# Relation Between Markers of Systemic Inflammation, White Matter Lesions, and Cognitive Performance in Late Adulthood

**DOI:** 10.1002/alz.087429

**Published:** 2025-01-09

**Authors:** Shivangi Jain, Alina Lesnovskaya, Lu Wan, Cristina Molina‐Hidalgo, Haiqing Huang, Anna Marsland, George Grove, Lauren Oberlin, Chaeryon Kang, Arthur Kramer, Charles Hillman, Edward McAuley, Jeffrey M Burns, Eric D Vidoni, Brad Sutton, Kirk I. Erickson

**Affiliations:** ^1^ AdventHealth Research Institute, Neuroscience, Orlando, FL USA; ^2^ University of Pittsburgh, Pittsburgh, PA USA; ^3^ Beckman Institute, Urbana, IL USA; ^4^ Northeastern University, Boston, MA USA; ^5^ University of Illinois, Urbana, IL USA; ^6^ University of Kansas Medical Center, Kansas City, KS USA

## Abstract

**Background:**

Aging is associated with heightened systemic inflammation, decline in selective aspects of cognition, and an increase in white matter lesions (WMLs). Both WMLs and systemic inflammation have been related to cognition. However, it is not clear how they interdependently relate to cognitive aging. In this study we examined whether WMLs mediate the relation between markers of systemic inflammation and cognition in late adulthood.

**Method:**

Baseline data from the randomized clinical trial “Investigating Gains in Neurocognition in an Intervention Trial of Exercise” (IGNITE) were used, which included 598 healthy older adults (mean age=69.9±3.75 years). Interleukin 6 (IL‐6) and Tumor Necrosis Factor Alpha (TNF‐α) were included as markers of systemic inflammation. A confirmatory factor analysis of cognitive performance resulted in factors measuring episodic memory (EM), processing speed (PS), working memory (WM), attentional control (AC), and visuospatial function (VF). WML volumes were computed from FLAIR and T1 scans using UBO detector. Mediation models were run with age, sex, years of education, and study site included as covariates.

**Result:**

Higher levels of IL‐6 were associated with more WMLs (b = 0.12, p < 0.001) as well as lower scores on all cognitive factors (all p < 0.03). In contrast, higher levels of TNF‐ α were associated with more WMLs (b = 0.10, p < 0.05) but not with any of the cognitive measures (all p > 0.3). More WMLs were associated with poorer PS, WM, AC, and VF (all p < 0.03), but not EM (all p > 0.05). Further, associations between IL‐6 and measures of PS, WM, AC, and VF were statistically mediated by WMLs (all b = ‐0.01, all p < 0.05).

**Conclusion:**

These findings suggest that markers of systemic inflammation might lead to poorer cognitive performance by impacting WMLs. The differences between IL‐6 and TNF‐α with cognition highlight the need for more research to understand how different cytokine pathways relate to cognition. Our results are important for building a mechanistic framework to understand the interplay between inflammation, WMLs, and cognition and could inform the development of targeted interventions focusing on regulating cytokines to support brain health and cognitive aging.

